# Supplementary material for: Degradative Effect of Nattokinase on Spike Protein of SARS-CoV-2
Source: Molecules. 2022 Aug 24;27(17):5405. doi: 10.3390/molecules27175405 (PMC9458005; doi:10.3390/molecules27175405)
Supplement: Supplementary file 1 [file molecules-27-05405-s001.zip › molecules-1840459-supplementary.pdf]

molecules
MDPI

Supplementary Information

Degradative Effect of Nattokinase on Spike Protein of SARS-CoV-2

Takashi Tanikawa [1,*,†], Yuka Kiba [2,†], James Yu [3], Kate Hsu [3], Shinder Chen [3], Ayako Ishii [4], Takami Yokogawa [2], Ryuichiro Suzuki [5], Yutaka Inoue [1] and Masashi Kitamura [2,*]

[1] Laboratory of Nutri-Pharmacotherapeutics Management, School of Pharmacy, Faculty of Pharmacy and Pharmaceutical Sciences, Josai University, Saitama 350-0295, Japan
[2] Laboratory of Pharmacognocy, School of Pharmacy, Faculty of Pharmacy and Pharmaceutical Sciences, Josai University, Saitama 350-0295, Japan
[3] Contek Life Science Co., Ltd., Taipei City 100007, Taiwan
[4] CellMark Japan, Tokyo 102-0071, Japan; ayako.ishii@cellmark.com
[5] Laboratory of Natural Products & Phytochemistry, Department of Pharmaceutical Sciences, Faculty of Pharmacy and Pharmaceutical Sciences, Josai University, Saitama 350-0295, Japan
* Correspondence: tanikawa@josai.ac.jp (T.T.); kitamura@josai.ac.jp (M.K.)
† These authors contributed equally to this work.

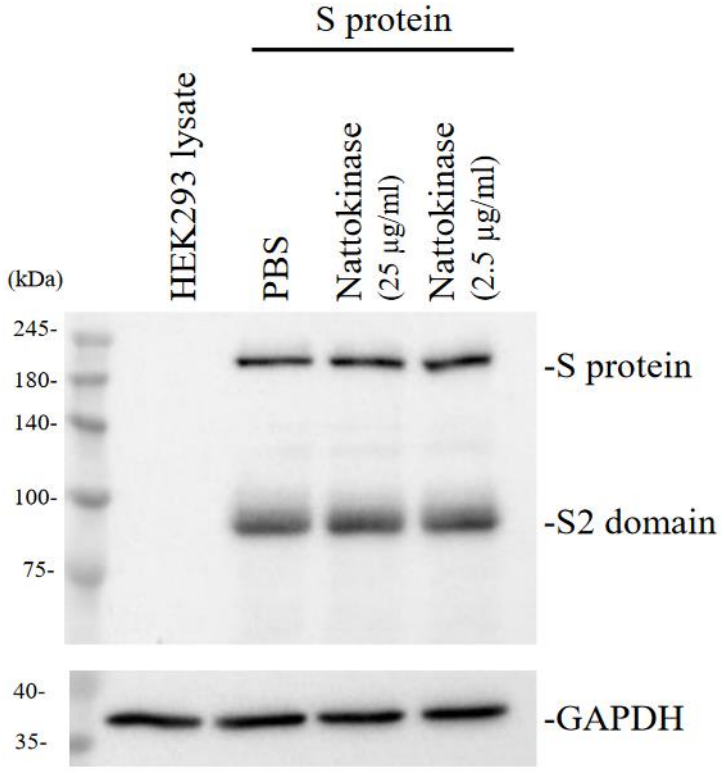

Figure S1. Effect of nattokinase addition to S protein expressing cell culture medium.was evaluated by westernblotting analysis. HEK293 cells were cultured at a density of3.5 × 105 cells/ml in DMEM supplemented with 10% FBS, L-glutamine, 100 U/mlpenicillin, and 100 µg/ml streptomycin. The cells were transfected with pcDNA3.1-SARS2-Spike and incubated for 9 h. After incubation, the cells were treated with nattokinase (25 and 2.5 µg/ml), incubated for 13 h. Western blotting was performed using anti-rhodopsin (C9) mouse monoclonal antibody (1D4) (Santa Cruz Biotechnology, TX, USA), anti-GAPDH mouse monoclonal antibody (FUJIFILM Wako)

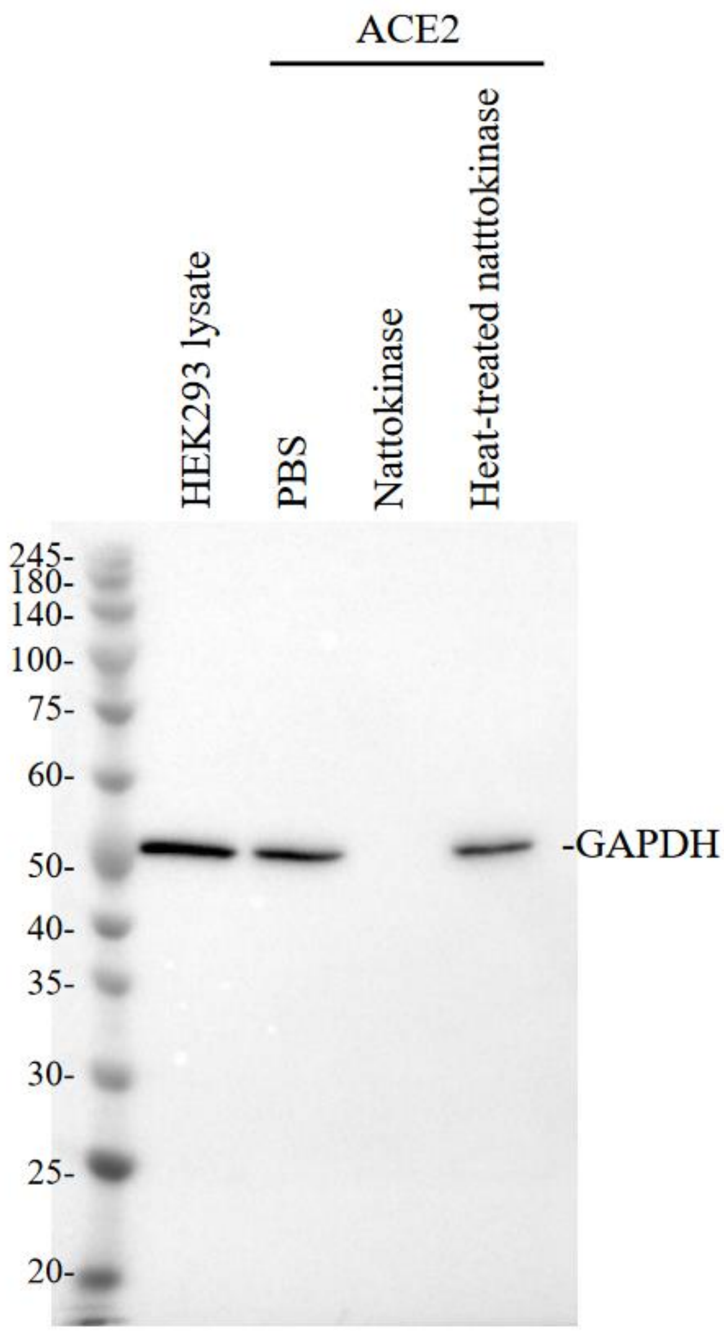

Figure S2. Degradative effects of GAPDH in vitro. The HEK293 cells were transfected with pcDNA3.1-hACE2 and incubated for 22 h. After incubation, the cultured cells were scraped and washed with ice-cold Dulbecco's phosphate-buffered saline (D-PBS). Cell lysates were incubated with nattokinase (7.5 μg/ml) and heat-treated nattokinase (7.5 μg/ml) and western blotting were performed. Equal volumes of the reaction mixture were loaded and western blotting was performed. The primary antibodies included anti-GAPDH mouse monoclonal antibody (FUJIFILM Wako) and secondaryantibodies include HRP-conjugated goat anti-mouse antibody (Proteintech).
